# Composition, Diversity, and Origin of the Bacterial Community in Grass Carp Intestine

**DOI:** 10.1371/journal.pone.0030440

**Published:** 2012-02-20

**Authors:** Shangong Wu, Guitang Wang, Esther R. Angert, Weiwei Wang, Wenxiang Li, Hong Zou

**Affiliations:** 1 The Key Laboratory of Aquatic Biodiversity and Conservation of Chinese Academy of Sciences, and State Key Laboratory of Freshwater Ecology and Biotechnology, Institute of Hydrobiology, Chinese Academy of Sciences, Wuhan, People's Republic of China; 2 Department of Microbiology, Cornell University, Ithaca, New York, United States of America; Charité-University Medicine Berlin, Germany

## Abstract

Gut microbiota has become an integral component of the host, and received increasing attention. However, for many domestic animals, information on the microbiota is insufficient and more effort should be exerted to manage the gastrointestinal bacterial community. Understanding the factors that influence the composition of microbial community in the host alimentary canal is essential to manage or improve the microbial community composition. In the present study, 16S rRNA gene sequence-based comparisons of the bacterial communities in the grass carp (*Ctenopharyngodon idellus*) intestinal contents and fish culture-associated environments are performed. The results show that the fish intestinal microbiota harbors many cellulose-decomposing bacteria, including sequences related to *Anoxybacillus*, *Leuconostoc*, *Clostridium*, *Actinomyces*, and *Citrobacter*. The most abundant bacterial operational taxonomic units (OTUs) in the grass carp intestinal content are those related to feed digestion. In addition, the potential pathogens and probiotics are important members of the intestinal microbiota. Further analyses show that grass carp intestine holds a core microbiota composed of Proteobacteria, Firmicutes, and Actinobacteria. The comparison analyses reveal that the bacterial community in the intestinal contents is most similar to those from the culture water and sediment. However, feed also plays significant influence on the composition of gut microbiota.

## Introduction

The gastrointestinal tract of a vertebrate is a complex ecosystem that often harbors a diverse bacterial community [1]. During the evolution of both the gut microbiota and the host, the epibiotic microbial community has become an integral component of the host and may affect the host biology [2], [3]. Among its many important functions, the gut microbiota can convert feedstuffs into microbial biomass and fermentation end products that can be utilized by the animal host [4], [5]. In the absence of this microbial fermentation, calories present in a diverse array of complex dietary glycans would be unavailable to the host [6]. The composition of the intestinal bacterial community is determined in part by dietary preferences and host life histories [2]. Cellulose, the major component of plant cell wall and the most common polysaccharide on earth, represents an important forage resource for herbivores. Ruminants are among of the most economically valuable herbivores and the microbial processes of the rumen have been extensively studied as improvements in cellulose degradation could have favorable impact on animal productivity. *Ruminococcus* and *Fibrobacter* species are important members of the rumen microbial community that enable the host to degrade and utilize fibrous plant materials efficiently as nutrients [7], [8], [9]. However, little is known about the associations between plant feed and the microbial communities in the digestive tract of aquatic animals, such as fish.

Gut microbiota may also play an important role in host health [10], [11]. In the absence of the gut microbiota, normal immune development and function are impaired. Further studies have shown that some symbiotic bacterial species, i.e. probiotics, may prevent inflammatory disease by not initiating an innate immune response during colonization [10], [12]. In addition to probiotics, the gut microbiota harbors opportunistic bacterial pathogens [13], [14]. The overgrowth of these pathogens may occur following a breach of intestinal microfloral barrier, which results from deficiencies in the host immune defense system or damage to the intestinal mucosal barrier [15], [16]. A comprehensive investigation of the normal microbiota associated with an animal will shed light on bacteria that help maintain healthy domestic animal stocks. However, surveys of normal intestinal microbiota have mainly focused on communities associated with terrestrial vertebrates [17], [18]. Current studies of fish intestinal bacteria are inadequate compared with those on terrestrial vertebrates.

The grass carp *Ctenopharyngodon idellus* is a native Chinese freshwater fish with a broad distribution in China, and has now been introduced to more than 100 countries [19]. The fish is widely cultivated for food. Production in China reached 4.08 million tons in 2009 and constitutes 21.4% of the total freshwater-cultured fish annual output [20]. Grass carp represents the largest freshwater aquaculture product in the world (ftp://ftp.fao.org/fi/stat/summary/a-6.pdf). Under natural conditions, the grass carp is basically herbivorous, feeding on certain aquatic weeds [21], [22]. When it feeds on aquatic plants, its daily ration (the relation of the total weight of feed taken in a day to the weight of the fish) may reach 49.9% [23]. However, with these high feeding rates feed materials pass through undigested and whole leaves are often found in the feces [22]. Elucidating the gut bacterial community composition and digestive processes of grass carp is essential for better management of the health and productivity of this important aquaculture species.

Microorganisms in the digestive tract of grass carp have been sporadically surveyed by several researcher groups. Based on conventional culture-dependent methods, researchers have detected pathogenic microorganisms in the intestinal tract [22], [24], found beneficial microbes that could enhance immunity and growth performance [24], [25], and revealed that *Vibrio* sp., *Aeromonas* sp., *Bacillus* sp., *Bacillus megaterium* and *Enterobacter asburiae* are major cellulose-degrading bacteria [26], [27], [28]. Recently, a 16S rRNA clone library analysis was performed to assess the bacterial diversity of the gut content of pond-reared grass carp [29]. Forty-eight operational taxonomic units (OTUs) were identified from gut contents, most affiliated with the Proteobacteria and Firmicutes. However, this could be an underestimation because rarefaction analysis showed that the sequencing approach was not carried out sufficiently to reach a plateau. Sufficient coverage of non-abundant and uncultured microbial groups in the gut sample requires deeper sequencing.

Understanding the factors that influence the composition of the microbial community in the fish alimentary canal is crucial in regulating the microflora, which will improve animal performance. Consequently, identification of the factors controlling the bacterial acquisition and community composition is of particular significance. Studies have demonstrated that fish have a distinct intestinal microflora compared with the external environment, and bacteria in the gut are generally those from the environment or diet [30], [31]. Research on grass carp has revealed that 75% of the OTUs, with a relative abundance ≥3% in the gut content, were also identified in feed and habitat samples [29]. However, so far, no further studies have been performed to determine which factors play more important roles in determining the fish intestinal microbiota.

The present study therefore aims to 1) characterize the intestinal bacterial community of grass carp, and 2) reveal the association between gut microbiota and microbiota from the associated environments. 16S rRNA gene fingerprinting methods [denaturing gradient gel electrophoresis (DGGE) and terminal restriction fragment length polymorphism (T-RFLP)] and bar-coded pyrosequencing are employed to determine the gut microbial communities of grass carp that feed on ryegrass, *Lolium perenne*, and the microbial communities of the related environments.

## Results

**Figure 1:**
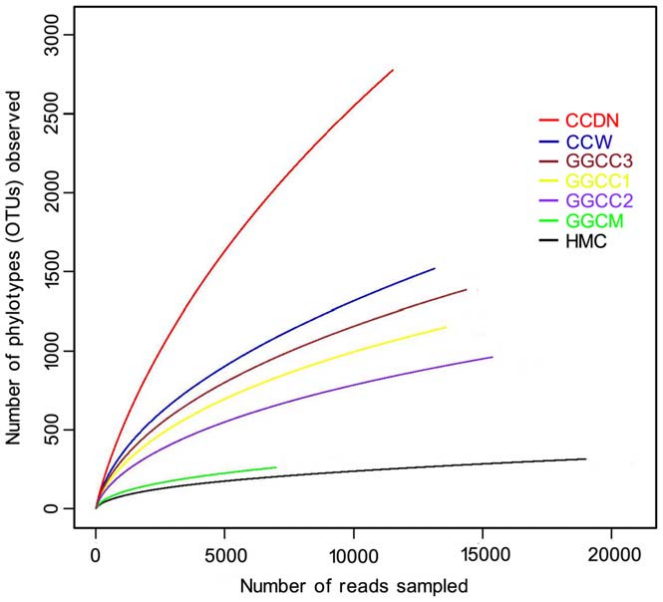
Rarefaction analysis of the different samples. Rarefaction curves of OTUs clustered at 97% sequence identity across different environmental samples.

A total of 93,991 valid reads and 6,058 OTUs were obtained from the seven samples through 454 pyrosequencing analysis, of which 67 reads and 48 OTUs were eukaryotes and were therefore excluded in the subsequent analyses. These sequences/OTUs were assigned to 25 different phyla or groups, and are available through the NCBI/EBI/DDBJ Short Read Archive (accession number ERA043547; http://www.ebi.ac.uk/ena/data/view/ERP000842). Each of the seven communities contained between 6990 and 18993 reads, with OTUs ranging from 259 to 2773. The rarefaction curves tended to approach the saturation plateau except in the CCDN (pond sediment) community (Fig. 1). Good's coverage estimations revealed that 94% to 98% of the species were obtained in all of the samples except for the CCDN sample wherein only 85% of the species were determined.

### Taxonomic composition

**Figure 2:**
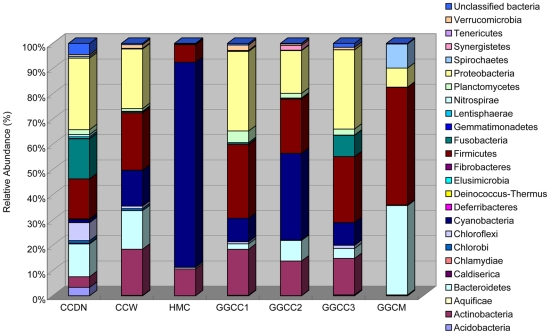
Bacterial composition of the different communities. Relative read abundance of different bacterial phyla within the different communities. Sequences that could not be classified into any known group were assigned as ‘Unclassified bacteria’.

All sequences were classified from phylum to genus according to the program Mothur using the default setting; 25 different phyla or groups were identified from these samples. The seven libraries showed very dissimilar 16S rRNA profiles even in phylum level distributions (Fig. 2). The CCDN library included the maximum number of phyla (24), where Proteobacteria, Firmicutes, Fusobacteria, Bacteroidetes, and Chloroflexi were the most important groups and accounted for 80.13% of the reads. The CCW (pond water) library was numerically dominated by Proteobacteria, Firmicutes, Actinobacteria, Bacteroidetes, and Cyanobacteria, and these phyla represented 94.04% of the reads. The HMC (grass carp feed, ryegrass) library showed relatively simple diversity, and Cyanobacteria, Actinobacteria, and Firmicutes represented 98.89% of the reads. The GGCC (GGCC represents GGCC1, GGCC2 and GGCC3; GGCC1, GGCC2 and GGCC3 stand for intestinal content of three different individuals of grass carp) libraries were dominated by Proteobacteria, Firmicutes, Cyanobacteria, and Actinobacteria, which accounted for 88.20%, 86.46%, and 80.97% of the reads in the GGCC1, GGCC2, and GGCC3 libraries, respectively. The GGCM (intestinal mucosa pooled from the three grass carp) library contained the lowest number of phyla (8), and reads from Firmicutes, Bacteroidetes, Spirochaetes, and Proteobacteria were the most abundant (99.08%).

The ten most abundant OTUs within the different samples were determined to understand further the important bacteria. The most abundant OTUs associated with the CCDN library were sequences related to *Prevotella* (5.51%), Fusobacteriales (2.21%–4.74%), *Veillonella* (4.13%–4.53%), *Dechloromonas* (2.54%), Sinobacteraceae (0.95%–1.81%), and *Streptococcus* (1.59%) (Table S1). The CCW library was dominated by sequences related to *Lactobacillus* (1.61%–7.65%) and *Flavobacterium* (3.87%), whereas the GGCC libraries were dominated by *Veillonella* (4.40%–12.57%), Methylocystaceae (2.21%–7.33%), Cyanobacteria (1.91%–27.28%), *Rothia* (3.83%–6.04%), *Streptococcus* (2.74%–5.37%), *Leuconostoc* (2.88%–3.87%), *Pseudomonas* (2.69%), *Anoxybacillus* (2.64%–2.80%), *Citrobacter* (1.84%), and *Clostridium* (1.74%) (Table S1). The most abundant sequences in the GGCM library were those related to Sphingobacteriales (9.23%–24.70%), *Clostridium* (11.89%–12.96%), and *Leuconostoc* (7.48%) (Table S1). For the HMC library, it was numerically dominated by sequences related to Cyanobacteria (0.79%–66.19%), *Actinomyces* (1.07%–3.04%), *Veillonella* (4.09%), and *Rothia* (2.3%) (Table S1).

The grass carp is herbivorous; therefore, cellulose-degrading bacteria are particularly important for food degradation, especially when feeding on a high-cellulose diet. In the present work, the following genera were abundant in the GGCC libraries: *Anoxybacillus*, *Leuconostoc*, *Clostridium*, *Actinomyces*, and *Citrobacter*. Of these genera, *Anoxybacillus* was abundant only in the GGCC3 community. *Actinomyces* was abundant not only in the HMC, but also in the GGCC communities. However, the *Actinomyces* OTUs abundant in the HMC community were few in the GGCC communities, and the *Actinomyces* OTUs abundant in the GGCC communities were absent in the HMC community (Table 1).

**Table 1:** The main cellulose-degrading bacteria present in the GGCC libraries.

Group	CCDN	CCW	GGCC1	GGCC2	GGCC3	GGCM	HMC
*Anoxybacillus*	0/0	0/0	45	20	851	2/2	0/0
*Leuconostoc*	0/1	0/2	596	516	274	561/565	0/0
*Clostridium*	1/19	3/9	612	239	13	2060/2123	0/0
*Actinomyces*	45/102	2/64	324	73	173	2/3	1013/1563
*Citrobacter*	2/2	1/1	479	142	15	140/140	0/0

Numbers below the diagonal line represent the total abundance of the genus presented in the community, whereas numbers above the diagonal line indicate the total abundance of all bacterial species (OTUs) shared between the corresponding sample and the GGCC libraries.

Grass carp suffers from many bacterial diseases, and studies have demonstrated that *Aeromonas caviae* causes bacterial enteritis and furunculosis, *Aeromonas sobria* causes peduncle disease, *Aeromonas hydrophila* is the pathogen of bacterial septicemia, *Pseudomonas fluorescens* is responsible for red skin disease, and *Flavobacterium columnare* is the etiology of columnaris disease and white head-mouth disease [22], [32]. In the present study, the distributions of these genera among the different samples were surveyed. The total reads of each genus within the CCDN, CCW, GGCM, and HMC libraries were counted, respectively; the read numbers of the OTUs common to GGCC libraries and to each environmental library were also calculated. Sequences related to *Aeromonas* were low in abundance in all libraries except the GGCM library (Fig. S1A). Sequences similar to *Pseudomonas* were most frequent among the different samples and the OTUs that occurred in the CCDN, HMC, and GGCM libraries were all present in the GGCC libraries (Fig. S1B). The sequences related to *Flavobacterium* had low abundance in the environmental libraries except the CCW library; however, they were common in the GGCC libraries (Fig. S1C).

*Bifidobacterium*, *Bacillus*, *Lactococcus*, and *Lactobacillus* are important inhabitants of the terrestrial vertebrate intestinal tract. In the current study, no reads related to *Bifidobacterium* was found among the seven libraries. Sequences similar to *Bacillus* were low in abundance in all samples (Fig. S2A). Sequences related to *Lactobacillus* were most abundant in the CCW library, up to 2725, and the OTUs shared between the CCW and GGCC libraries included 1381 reads in the CCW library. Meanwhile, the OTUs shared between the HMC and GGCC samples contained 175 sequences in the HMC library. However, only 2 to 17 reads were present in the GGCC1, GGCC2, and GGCC3 libraries (Fig. S2B). In contrast to that of *Lactobacillus*, few reads related to *Lactococcus* were found in the CCDN, CCW, and HMC communities, whereas highly abundant reads similar to *Lactococcus* occurred in the GGCC communities (Fig. S2C).

### Core intestinal microbiota

**Figure 3:**
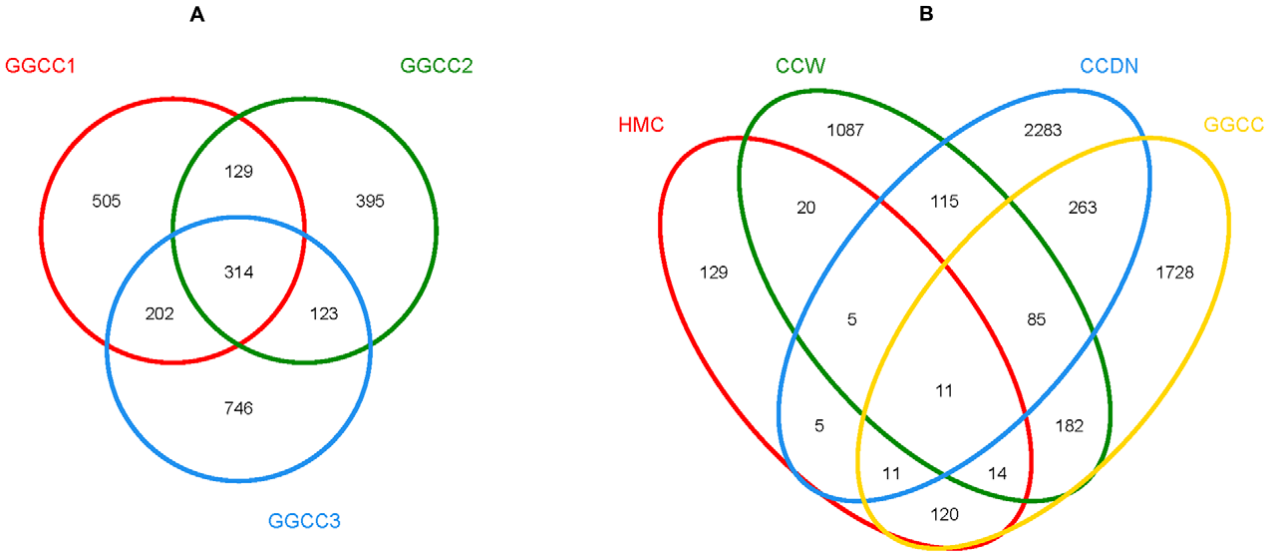
Shared OTU analysis of the different libraries. Venn diagram showing the unique and shared OTUs (3% distance level) in the different libraries (A) for the GGCC1, GGCC2, and GGCC3 libraries, and (B) for the HMC, CCW, CCDN, GGCM and GGCC libraries. * GGCC represents GGCC1, GGCC2 and GGCC3; GGCC1, GGCC2 and GGCC3 mean intestinal content of different individuals of grass carp. HMC, GGCM, CCDN, and CCW stand for grass carp feed ryegrass, intestinal mucosa of grass carp, pond sediment and pond water, respectively.

**Figure 4:**
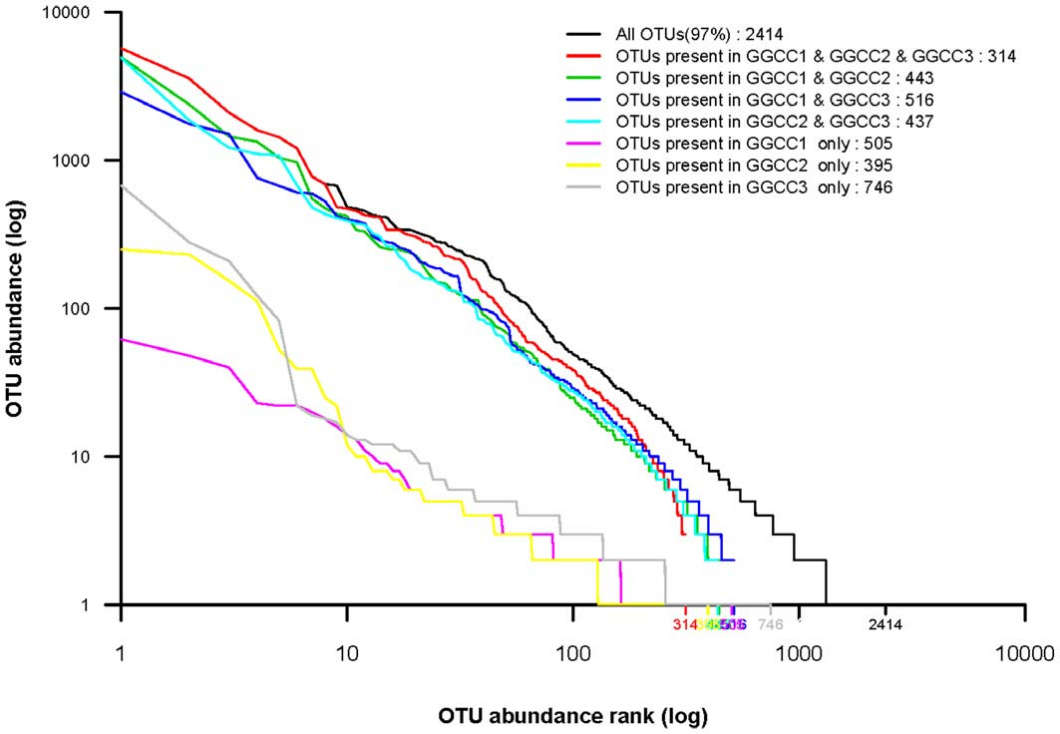
Rank abundance analysis of the different bacterial community groups. Rank abundance distribution curves showing the OTUs within each category of the Venn diagram in Fig. 3A ranked according to their abundance in the corresponding combined OTU sequence data set.

The bacterial species in the GGCC libraries were further investigated for the presence of a core gut microbiota. Figure 3A and Table 2 show that the GGCC1, GGCC2, and GGCC3 libraries have 314 OTUs in common. Species rank abundance distribution curves revealed that the OTUs present in all three libraries contained the most abundant OTUs in any library, whereas the OTUs observed in only one or two libraries tended to be relatively low in abundance (Fig. 4). Statistical analysis revealed that the OTUs common to the three libraries comprised 79.83%, 78.07%, and 69.35% of the reads in the GGCC1, GGCC2, and GGCC3 libraries, respectively (Table 2). Proteobacteria, Firmicutes, and Actinobacteria included 222 of the shared OTUs (70.93% in proportion), and 23647 shared reads (72.09% in proportion). Within these three phyla, Alphaproteobacteria, Gammaproteobacteria, Clostridia, Bacilli, and Actinobacteridae represented the most abundant classes common to the three libraries. For Bacteroides, only 5 OTUs were common to the GGCC1, GGCC2, and GGCC3 libraries, and they tended to be low in abundance (Table 2).

**Table 2:** Shared phyla among the GGCC libraries.

		*Shared reads*		
*Phylum*	*Shared OTUs*	*GGCC1*	*GGCC2*	*GGCC3*
*Acidobacteria*	*2*	*2*	*2*	*2*
***Actinobacteria***	*63*	*2017*	*1832*	*1592*
*Bacteroidetes*	*5*	*26*	*12*	*103*
*Chloroflexi*	*5*	*21*	*7*	*35*
*Cyanobacteria*	*46*	*1138*	*5100*	*1106*
***Firmicutes***	*55*	*3075*	*2694*	*3135*
*Fusobacteria*	*5*	*36*	*52*	*40*
*Planctomycetes*	*16*	*517*	*261*	*192*
***Proteobacteria***	*104*	*3763*	*1953*	*3586*
*Verrucomicrobia*	*7*	*233*	*79*	*76*
*Unclassified*	*6*	*23*	*12*	*80*
*Total shared sequences*	*314*	*10851*	*12004*	*9947*
*Total reads*		*13593*	*15376*	*14344*
*Shared reads/Total reads (%)*		*79.83*	*78.07*	*69.35*

The phyla in bold letters represent core gut microbiota.

### Relationships between bacterial communities in the fish intestinal content and fish-associated environmental bacterial communities

**Figure 5:**
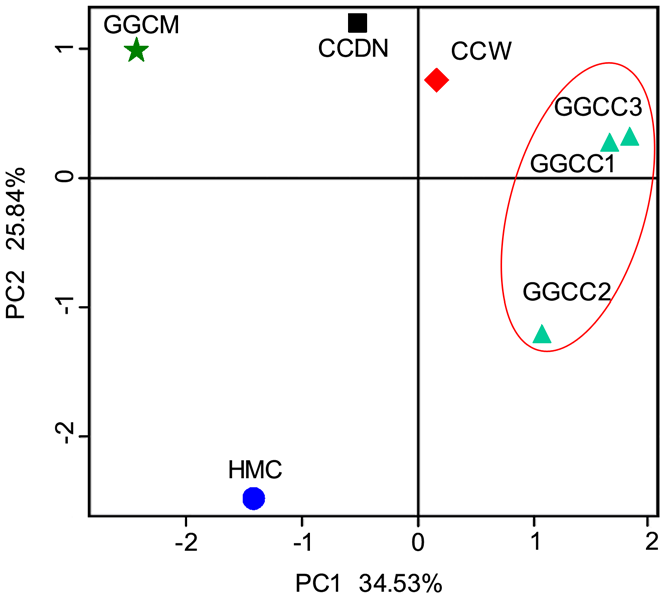
Sample Sorting analysis. Scatterplot of PCA-score depicting variance of fingerprints derived from different bacterial community. Principal components (PCs) 1 and 2 explained 34.53% and 25.84% of the variance, respectively.

**Figure 6:**
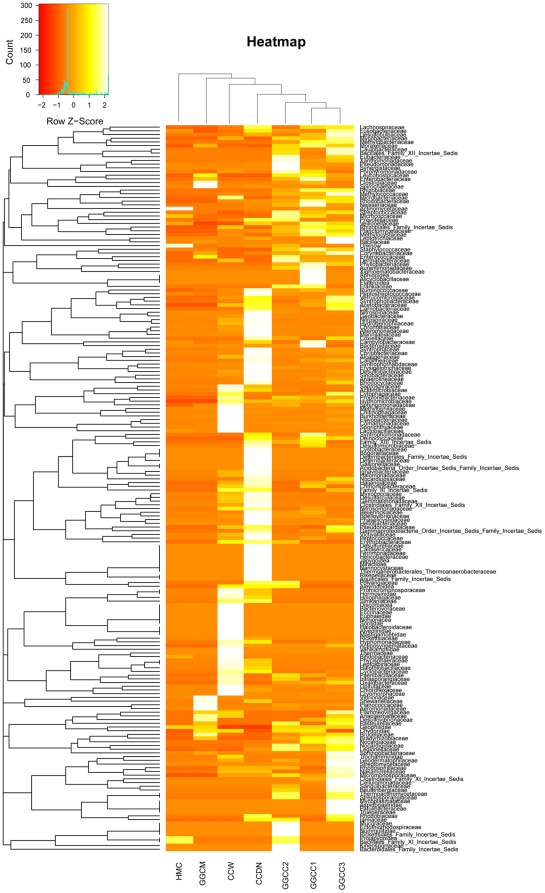
Bacterial distribution among the seven samples. Double hierarchical dendrogram showing the bacterial distribution among the seven samples. The bacterial phylogenetic tree was calculated using the neighbor-joining method and the relationship among samples was determined by Bray distance and the complete clustering method. The heatmap plot depicts the relative percentage of each bacterial family (variables clustering on the Y-axis) within each sample (X-axis clustering). The relative values for bacterial family are depicted by color intensity with the legend indicated at the bottom of the figure. Clusters based on the distance of the seven samples along the X-axis and the bacterial families along the Y-axis are indicated in the upper and left of the figure, respectively.

The DGGE profile reveals that the GGCC communities have predominant DGGE bands that are different from those of the environmental samples. The hierarchical cluster analysis using MVSP 3.1 software showed that the GGCC and GGCM communities grouped together, and then clustered with the CCDN, CCW, and HMC communities in order (Fig. S3). This clustering result is supported by the T-RFLP analysis (data not shown). The principal component analysis with the weighted UniFrac distance and heatmap analysis were determined using pyrosequencing data to corroborate further the findings from these DNA fingerprinting methods. The principal component analysis (PCA) score plot revealed that the GGCC communities harbored characteristic bacterial communities, and all of the GGCC samples grouped to the right of the graph along PC1, which accounts for 34.53% of the total variations. The CCW and CCDN samples were closely related to the GGCC samples, whereas the HMC sample was separate from the other samples along PC2, which represented 25.84% of the total variations (Fig. 5). Overall, the two PCA axes explained 60.37% of the variation between the different communities. Hierarchically clustered heatmap analysis based on the bacterial community profiles at family level disclosed that GGCC samples grouped together firstly, and they then clustered with CCDN, CCW, GGCM, and HMC samples in order (Fig. 6). In addition, the ∫-LIBSHUFF analyses indicated that the GGCC samples were significantly different from the GGCM sample (GGCC1, GGCC2 or GGCC3 library vs. GGCM library; each *p* value = 0).

The species shared among these communities were determined via a Venn diagram to compare the relationships among these communities in detail. The results show that the number of species shared between the GGCC and HMC communities was 156 (Fig. 3B), i.e., 50% of the OTUs in the HMC library were present in the GGCC libraries. The most abundant OTUs shared by the two groups were *Veillonella* (4.09% and 8.25% of the HMC and GGCC reads, respectively) and *Rothia* (2.30% and 3.70% of the HMC and GGCC reads, respectively), except for two OTUs related to Cyanobacteria. The GGCC and CCW communities had 292 OTUs in common (Fig. 3B), of which Methylocystaceae (0.64% and 4.85% of the CCW and GGCC reads, respectively), *Rhodobacter* (0.15% and 0.78 of the CCW and GGCC reads, respectively), and Methylococcaceae (*Methylocaldum*) (0.21% and 1.24% of the CCW and GGCC reads, respectively) were in both communities. The GGCC and CCDN libraries shared the most OTUs (370), and the most abundant OTU shared by the two communities were *Veillonella* (4.13% and 8.25% of the CCDN and GGCC libraries, respectively).

## Discussion

Complex intestinal microbial communities are believed to provide some benefits to their host [4], and have now received increasing attention. Thus far, studies regarding fish intestinal microbial flora are relatively limited, especially in relation to the microbiota of herbivorous fish. In the current study, the microbial community of grass carp intestinal tract has been determined in detail and the origins of the prominent intestinal populations have been investigated. The present work represents the first implementation of second-generation sequencing technology for investigating the intestinal microbial community of fish with economic importance.

Surveys on the vertebrate gut samples, especially terrestrial mammalian gut microbiota, suggest that Firmicutes and Bacteroidetes are numerically the most dominant phyla [2], [33]. However, the present study suggests that in the GGCC libraries, Proteobacteria is the most abundant, followed by Firmicutes. The abundance of Bacteroidetes is relatively low. The incongruence may probably be due to the differences in host and the host living conditions; Ley et al. [2] and Qin et al. [33] based their conclusions mainly from that of terrestrial mammals, while in this study grass carp is aquatic vertebrate. The results, however, are generally consistent with those of Han et al. [29], Wu et al. [13], and Roeselers et al. [14] who demonstrated that Proteobacteria and Firmicutes were the most ubiquitous and common, and Bacteroidetes was relatively low in the intestinal contents of different fishes.

Surprisingly, Actinobacteria were prevalent members of the intestinal bacterial communities and they were more abundant than Bacteriodetes in the present study. Actinobacteria are well known for production of secondary metabolites, of which many are potent antibiotics [34]. Actinobacteria are widely distributed in both terrestrial and aquatic (including marine) ecosystems, especially in soil, where they play a crucial role in the recycling of refractory biomaterials through decomposition and humus formation [34]. However, the phylum generally makes up a small proportion of bacterial sequences retrieved from host intestine [2], [35]. Only a limited number of studies have reported Actinobacteria as the dominant microbiota in the vertebrate gut, e.g., the sheep hindgut and fishes. [13], [29], [36], [37]. PCR biases were evaluated to determine the reason for this discrepancy. The comparison with the RDP databases show that the primers used in the current study are more sensitive for Bacteroidetes than for Actinobacteria (Table S2). The results indicate that Actinobacteria are naturally more abundant than Bacteroidetes in the fish intestines, at least, in grass carp. Venn diagram and Rank abundance distribution curves suggest that the Actinobacteria in the GGCC communities may have mainly originated from the CCW community (data not shown).

Cellulose-decomposing bacteria have been extensively studied over recent decades [38]. In these studies, the predominant cellulose-degrading bacteria are different in dissimilar environments. Bacilli (the phylogenetic group Firmicutes) or *Cytophaga*-like bacteria (the Bacteroidetes group) are the main agents responsible for bacterial cellulose degradation in eutrophic habitats with neutral pH, whereas Actinobacteria appear dominant in aerobic cellulose degradation in *Sphagnum* peat bogs under acidic conditions (pH 3.5–5.5) [39]. *Ruminococcus* spp. and *Fibrobacter* spp. are the main cellulose-decomposing bacteria in the rumen [5], [9], [40]. In culture-based studies, *Bacillus*, *Vibrio*, *Aeromonas*, and *Enterobacter* have been found to be cellulose-degrading microbes from grass carp intestines [26], [27]. In the current study, cellulase activity was not determined with respect to individual bacterial strains. However, a comprehensive analysis was performed to compare the intestinal bacteria with the published cellulose-degrading bacteria. The results indicate that the cellulose-degrading bacteria in grass carp intestine may be peculiar; *Anoxybacillus*, *Leuconostoc*, *Clostridium*, *Actinomyces*, and *Citrobacter* were abundant and they may represent the main cellulose-decomposing bacteria in this system. Further study reveals that these cellulose-degrading bacteria are of low abundance in CCDN, CCW and HMC libraries, indicating that these bacteria are enriched in the intestine by cellulosic feed. In addition, our results disclose that different individuals harbor varied populations of these potential cellulose degraders, which is consistent with the findings of Weimer [9] and of Koike and Kobayashi [41].

Of the 10 most abundant bacterial OTUs in grass carp intestinal contents, several are related to known cellulose-decomposing bacteria. In addition to cellulose-degrading bacteria, some OTUs recovered are related to *Streptococcus* and *Prevotella*. Species of the genus *Streptococcus* are active proteolytic rumen bacteria [42], and *Prevotella* species are prominent inhabitants of the rumen and play a central role in ruminal digestion of feed proteins [43]. These results are consistent with those of Rawls et al. [3] and Ley et al. [2] that intestinal bacteria play important roles in host energy metabolism.

The potential pathogens of serious bacterial diseases of the fish were also surveyed. The results reveal that these bacteria are ubiquitous in both the aquacultural environment and in fish intestine. Genera that contain the two most important opportunistic pathogens of grass carp, *Pseudomonas* and *Flavobacterium*, are highly abundant in the intestinal contents. This finding is in accordance with those of Pond et al. [44] and Wu et al. [13], who proposed that the fish digestive tract is a reservoir for many opportunistic pathogens. Although several *Aeromonas* spp. are potential pathogens, the present study reveals that *Aeromonas* is highly abundant in the intestinal mucosa of grass carp. *Aeromonas* spp. have been detected in the normal intestinal mucosa from several fishes, such as Arctic charr (*Salvelinus alpinus* L.), Atlantic cod (*Gadus morhua* L.) and zebrafish (*Danio rerio*) [14], [31], [45]. Bacteria in the mucosa may be regarded as indigenous species, and are involved in host nutrition, mucosal defense, and host immunity [11], [46]. The results support the studies by Gibson et al. [47], Gibson [48], and Irianto and Austin [49], which found that *Aeromonas* may play more important roles in fish biology, other than as pathogenic microbes. However, the highly abundant presence of *Aeromonas* in the mucosa may agree with the findings of Hiney et al. [50], Lødemel et al. [45], and Yang [51] that the intestine might be the primary location for *Aeromonas* colonization under stress-induced infections. Clearly additional studies are needed to determine the role of intestinal *Aeromonas* spp. in the grass carp.

Probiotics have been widely used in aquaculture [52], [53]. Probiotics may prevent pathogens from proliferating in the intestinal tract, and in the culture environment and may improve condition of the fish by securing optimal use of the feed, improving water quality, or stimulating the immune system of the host [52]. The fish intestinal microbiota might be a key pool of potential probiotics for cultured fish species [29]. Previous studies have indicated that lactic acid bacteria (*Lactobacillus*, *Streptococcus*, and *Lactococcus*), *Bacillus*, and *Pseudomonas* are important biological control agents in aquaculture [53], and these bacteria have been detected in the intestine of grass carp and are candidate probiotics. However, the present study shows that *Lactobacillus* species have low abundance in the intestine, although they were common in the CCW library. This finding suggests that *Lactobacillus* species may not serve as effective probiotics in this system, as the lactic acid bacteria cannot establish large populations in the grass carp intestine. This finding, to a certain extent, interprets that the added lactic acid bacteria through food or feed preparations show a sharp decrease and are lost from the gastrointestinal tract in most of the fish within a few days after the intake stopped [52], [54]. *Bacillus* species were present with low abundance in the culture system in this study, which is inconsistent with previous results [26]. We suggest that the difference may arise from primer bias, as the primers used in this study are insensitive compared with other primers (Table S2). Although, *Pseudomonas* were highly abundant in the intestinal community, which may imply that this group plays important functions and are potential probiotics, they should be considered with caution because some *Pseudomonas* spp. are potential pathogens. *Bifidobacterium* are ubiquitous in mammalian intestine and are beneficial to the host [55]. However, in the current study, members of this group were not found in the grass carp intestine and therefore are not likely candidates for probiotic use. Future studies should evaluate the abundance and retention of the putative probiotics in the fish intestine.

Specific intestinal microbiota have been widely recognized [52], and the concept of core intestinal microbiota has been proposed in the context of mammalian hosts [33], [56]. Recently, this model has been applied to teleost fishes [14], [29]. In the present study, PCA and heatmap plots of the bacterial communities derived from grass carp, and the 16S rRNA gene fingerprinting based analyses, suggest that fishes harbor specific intestinal microbiota. Previous studies have shown that Proteobacteria, Firmicutes, and Actinobacteria are dominant in the fish intestinal content using conventional culture-dependent methods or conventional molecular techniques [29], [57]. In addition, Bacteroides has also been found to be important bacterial members in grass carp intestine [29], [57], [58]. In this study, Proteobacteria, Firmicutes, Cyanobacteria, and Actinobacteria dominated the GGCC libraries, and accounted for 88.20%, 86.46% and 80.97% of the reads in the GGCC1, GGCC2, and GGCC3 libraries, respectively. The three libraries had 314 OTUs in common, which comprised 79.83%, 78.07% and 69.35% of the reads of GGCC1, GGCC2, and GGCC3 libraries, respectively. Proteobacteria, Firmicutes, and Actinobacteria included 222 shared OTUs (70.93% in proportion), and 23647 shared reads (72.09% in proportion). However, the number of OTUs and OTU abundance of the Bacteroides common to the GGCC1, GGCC2, and GGCC3 libraries were low. The data and previous studies indicate that Proteobacteria, Firmicutes, and Actinobacteria comprise the bacterial core set of the intestinal content of grass carp.

Previous investigations have proposed that the gastrointestinal microbiota of fish originate from their environment [29], [30]. In the present study, pyrosequencing was used to evaluate the potential origin of the gut bacteria by determining if similar sequences were located in the fish environment. Both the PCA and the heatmap analyses show that the gut bacterial communities are more similar to the CCW and CCDN libraries than the HMC (feed) library. In addition, the Venn diagram indicates that the GGCC libraries shared more species with the CCW and CCDN libraries than the HMC library. These results suggest that the intestinal bacteria of grass carp may mainly be from the water and sediment. In terms of feed, the HMC library is distant from the GGCC libraries; however, 50% of the OTUs in the HMC library were present in the GGCC libraries, indicating that feed may significantly influence the composition of the gut microbiota.

## Materials and Methods

### Sample collection

The grass carp was raised in an artificial pond in Jingzhou City, Hubei Province, China from October 6 to December 2, 2010. The pond is located in the middle reaches of Yangtze River, where is the major producing region of the fish. The water depth and coverage of the pond are approximately 1.5 m and 100 square meters, respectively. The fish were added specifically for this experiment and were cultured from fry. Our postgraduate took care of the fish while they were in the pond. During the experimental period, the fish were fed ryegrass, which is widely used in the culture of this fish species; the fish were fed to apparent satiation twice a day (09:30, 15:30 h). At the end of the feeding experiment, three fish with an average weight of approximately 900 g were harvested with nets and sacrificed. The fish were chosen by chance, and more than three fish were caught so the other fish were sent back to the pond. The fish were then euthanized in the laboratory through washrag soaked with MS-222. Sampled fish were dissected immediately with sterile scissors. The intestines were aseptically removed from their abdominal cavity and the contents were gently squeezed out and harvested, separately. Thereafter, the epithelial intestinal mucosa of the three fish were collected and pooled together as described elsewhere [31]. In addition, water and surface sediment samples were separately collected from 3 locations in the same pond and pooled together. Pond water was sampled at a depth of approximately 50 cm to the top water layer. Microorganisms present in the sample were collected by filtration of 250 mL of water onto 0.2 µm pore size hydrophilic polyethersulfone membrane filter (47 mm diameter, Pall, Lane Cove, Australia). Sediment samples were sampled using a Petersen grab, and only the unconsolidated surface sediments were collected. Samples of the fish feed, ryegrass planted aside the pond, were also taken. The sampling location and procedures are provided in detail in figure S4. All the samples were placed into sterile polypropylene centrifuge tubes (BD Falcon, BD Biosciences) and stored provisionally in a portable refrigerator at −20°C, and transferred to laboratory within 24 hours and kept frozen at −80°C until DNA extraction.

### Ethics statement

No specific permits were required for the described field studies. No specific permissions were required for the artificial pond in Jingzhou City, Hubei Province, China. It is not privately-owned or protected in any way. The field studies did not involve endangered or protected species. This study has been reviewed and approved by the ethics committee of the Institute of Hydrobiology, Chinese Academy of Sciences.

### DNA Extraction and Purification

Samples (180 mg of sediment, intestinal content, or mucosa; filters from 250 mL water) were suspended in 1400 µL of ASL buffer, and genomic DNA was extracted using a QIAamp® DNA Stool Mini Kit (Qiagen, Germany) with slight modification. Sterile zirconia beads were added to the samples, which improves extraction yield and the quality of the community DNA [59]. For each sample, DNA was extracted in duplicate to avoid bias [13], and the extracts from the same sample were pooled. DNA purity and concentration was analyzed spectrophotometrically using the e-Spect ES-2 (Malcom, Japan). The extracted DNA was stored at −20°C until use.

### PCR amplification, DGGE and T-RFLP analyses

For DGGE analysis, a nested PCR was performed using the following general bacterial primer combinations: 27F-1492R [60] and 968F-1401R (secondary) [61]. DGGE profiling was performed on a Dcode universal mutation detection system (Bio-Rad laboratories Inc., USA) according to the manufacturer's instructions. For T-RFLP analysis, the PCR primers (27F and 1492R) were used, and the primer 27F was fluorescently labeled on its 5′-end with carboxifluorescein (5′-/6-FAM). Three restriction endonucleases, *Hae*III (15 U), *Msp*I (10 U), or HhaI (10 U) (Fermentas, China), were used. The digested fragments were separated on a 3730 DNA Analyzer (Applied Biosystems, USA). The details of PCR amplification, DGGE and T-RFLP analyses are in the Text S1.

### PCR amplification, amplicon quantitation, pooling, and pyrosequencing

A region ∼526 bp in the 16S rRNA gene, covering the V1–V3 region was selected to construct community library through tag pyrosequencing. The bar-coded broadly conserved primers 27F and 533R containing the A and B sequencing adaptors (454 Life Sciences) were used to amplify this region. The forward primer (B-27F) was 5′-*CCTATCCCCTGTGTGCCTTGGCAGTCTCAG*AGAGTTTGATCCTGGCTCAG -3′, where the sequence of the B adaptor is shown in italics and underlined. The reverse primer (A-533R) was 5′-*CCATCTCATCCCTGCGTGTCTCCGACTCAGNN*NNNNNNNNTTACCGCGGCTGCTGGCAC-3′, where the sequence of the A adaptor is shown in italics and underlined and the Ns represent an eight-base sample specific barcode sequence. The length of the amplicon, including the barcode and 454 primers, was ∼596 nt. The PCRs were carried out in triplicate 50 µL reactions with 0.6 µM each of the primer, ∼5 ng of template DNA, and 1× PCR reaction buffer, 2.5 U of Pfu DNA Polymerase (MBI. Fermentas, USA). The amplification program consisted of an initial denaturation step at 94°C for 4 min, followed by 25 cycles, where 1 cycle consisted of 94°C for 30 s (denaturation), 55°C for 30 s (annealing) and 72°C for 30 s (extension), and a final extension of 72°C for 10 min. During amplification, negative controls were also performed. Replicate PCR products of the same sample were assembled within a PCR tube. Then they were visualized on agarose gels (2% in TBE buffer) containing ethidium bromide, and purified with a DNA gel extraction kit (Axygen, China).

Prior to sequencing, the DNA concentration of each PCR product was determined using a Quant-iT PicoGreen double-stranded DNA assay (Invitrogen, Germany) and was quality controlled on an Agilent 2100 bioanalyzer (Agilent, USA). Following quantitation, the amplicons from each reaction mixture were pooled in equimolar ratios based on concentration and subjected to emulsion PCR to generate amplicon libraries, as recommended by 454 Life Sciences. Amplicon pyrosequencing was performed from the A-end using a 454/Roche A sequencing primer kit on a Roche Genome Sequencer GS FLX Titanium platform at Majorbio Bio-Pharm Technology Co., Ltd., Shanghai, China.

### Statistical and bioinformatics analysis

The presence/absence of T-RFs or DGGE bands was exported to generate a matrix. Clustering analyses based on the T-RFLP or DGGE profile of different samples were performed with the program MVSP 3.1 [62]. The Jaccard's similarity coefficients were calculated for the clustering.

Considering previous studies described sources of errors in 454 sequencing runs, the valid reads should comply with the following rules: each pyrosequencing read containing a primer sequence should be 350–600 bp in length, have no ambiguous bases, match the primer and one of the used barcode sequences, and present at least an 80% match to a previously determined 16S rRNA gene sequence. These pyrosequencing reads were simplified using the ‘unique.seqs’ command to generate a unique set of sequences, and then were aligned using the ‘align.seqs’ command and compared with the Bacterial SILVA database (SILVA version 106; http://www.arb-silva.de/documentation/background/release-106/). The aligned sequences were further trimmed and the redundant reads were eliminated using the ‘screen.seqs’, ‘filter.seqs’, and ‘unique.seqs’ commands in order. The ‘chimera.slayer’ command was used to determine chimeric sequences. The ‘dist.seqs’ command was performed, and unique sequences were clustered into OTUs defined by 97% similarity. Rarefaction analysis and Good's coverage for the seven libraries were determined; heatmap figures, Venn diagrams, and species rank abundance distribution curves (Whittaker plots) were generated using custom Perl scripts; and ∫-LIBSHUFF analysis was performed using the libshuff command. In addition, a principal component analysis (PCA) was performed based on weighted UniFrac distance. In the present study, data preprocessing, OTU-based analysis, and hypothesis testing were performed on Mothur [63].

## Supporting Information

Figure S1 **Distribution of pathogenic microorganisms.** Distribution of main pathogenic microorganisms among different samples (A) *Aeromonas*, (B) *Pseudomonas*, and (C) *Flavobacterium*. The dark red column indicates the total abundance of all bacterial species shared between the corresponding sample and the GGCC libraries, whereas the sky blue histogram represents the total abundance of the genus presented in the community. In addition, the read numbers on Y-axis were log 10-transformed before plotting.
(TIF)

Figure S2 **Distribution of probiotics.** Distribution of main probiotics among different samples (A) *Bacillus*, (B) *Lactobacillus*, and (C) *Lactococcus*. Dark red column means total abundance of all bacterial species shared between corresponding sample and GGCC libraries, while sky blue histogram represents total abundance of the genus presented in the community. In addition, read numbers on Y-axis were log 10-transformed before plotting.
(TIF)

Figure S3 **bacteria community similarity analysis.** Comparison of bacteria community similarity based on DGGE fingerprint of 16S rRNA sequences.
(TIF)

Figure S4 **Sampling locality and procedures.** Sampling locality and procedures in the present study. (A) Sampling locality, (B) Sampling procedures.
(TIF)

Table S1 **Classification of the 10 most abundant bacterial OTUs in the grass carp intestine contents and the associated environment, listed from most to least abundant.** Relative abundance (%) of each OTU is included in parentheses. OTUs were identified using 97% cutoffs. GGCC1, GGCC2 and GGCC3 mean intestinal content of different individuals of grass carp. HMC, GGCM, CCDN, and CCW stand for grass carp feed ryegrass, intestinal mucosa of grass carp, pond sediment and pond water, respectively.
(DOC)

Table S2 **Primer coverage.** The primer sequences were compared with the RDP 16S rRNA gene sequence database to examine primer coverage using the Probe Match tool (http://rdp.cme.msu.edu/probematch/search.jsp) and exact matches were counted. 27F (used in this study), 5′-AGAGTTTGATCCTGGCTCAG-3′ 533R (used in this study), 5′- TTACCGCGGCTGCTGGCAC-3′ 534R, 5′-CAATTACCGCGGCTGCTGG-3′ 338R, 5′-TGCTGCCTCCCGTAGGAGT-3′ 338F, 5′-ACTCCTACGGGAGGCAGCAG-3′ 518R, 5′-ATTACCGCGGCTGCTGG-3′ 784F, 5′-AGGATTAGATACCCTGGTA-3′ 1061R, 5′-CRRCACGAGCTGACGAC-3′ (* R = A/G).
(DOC)

Text S1 **Protocol of PCR amplification, DGGE and T-RFLP analyses.**
(DOC)
