# Supplementary figures and images for: Composition, Diversity, and Origin of the Bacterial Community in Grass Carp Intestine
Source: PLoS One. 2012 Feb 20;7(2):e30440. doi: 10.1371/journal.pone.0030440 (PMC3282688; doi:10.1371/journal.pone.0030440)

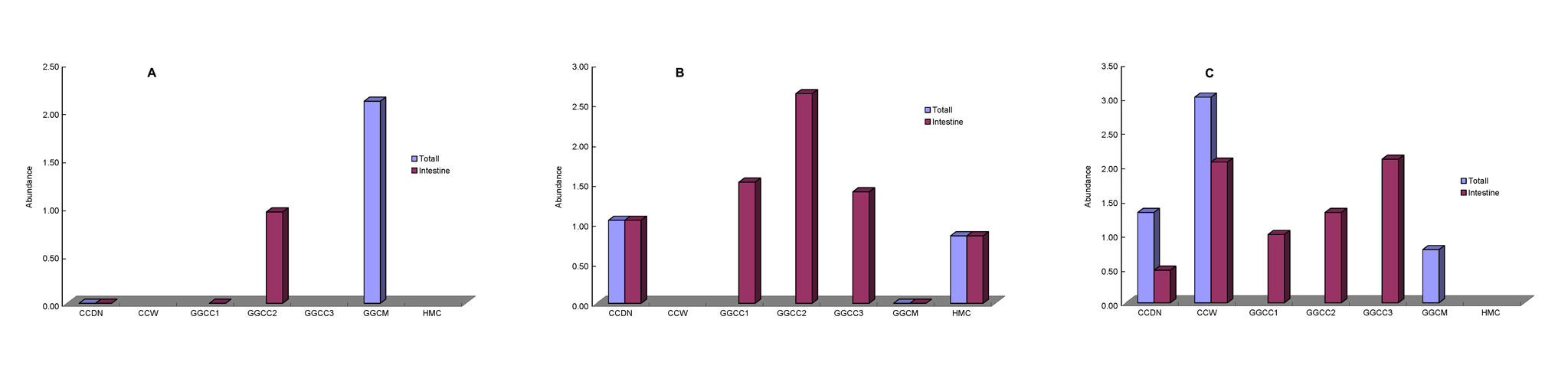

Supplement: Figure S1 — Distribution of pathogenic microorganisms. Distribution of main pathogenic microorganisms among different samples (A) Aeromonas, (B) Pseudomonas, and (C) Flavobacterium. The dark red column indicates the total abundance of all bacterial species shared between the corresponding sample and the GGCC libraries, whereas the sky blue histogram represents the total abundance of the genus presented in the community. In addition, the read numbers on Y-axis were log 10-transformed before plotting. (TIF) [file pone.0030440.s001.tif]

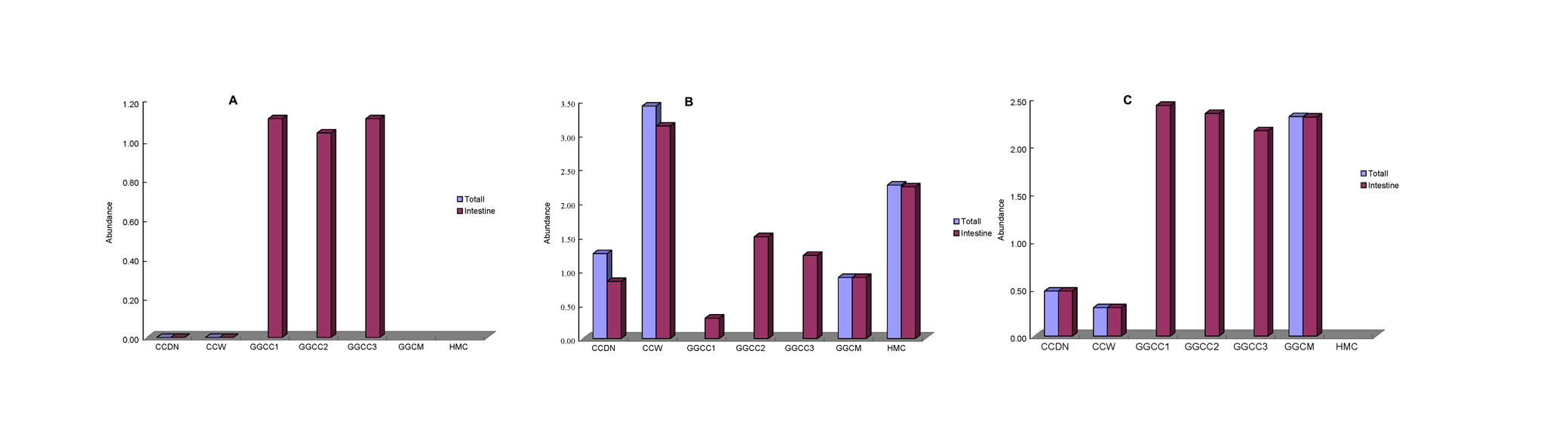

Supplement: Figure S2 — Distribution of probiotics. Distribution of main probiotics among different samples (A) Bacillus, (B) Lactobacillus, and (C) Lactococcus. Dark red column means total abundance of all bacterial species shared between corresponding sample and GGCC libraries, while sky blue histogram represents total abundance of the genus presented in the community. In addition, read numbers on Y-axis were log 10-transformed before plotting. (TIF) [file pone.0030440.s002.tif]

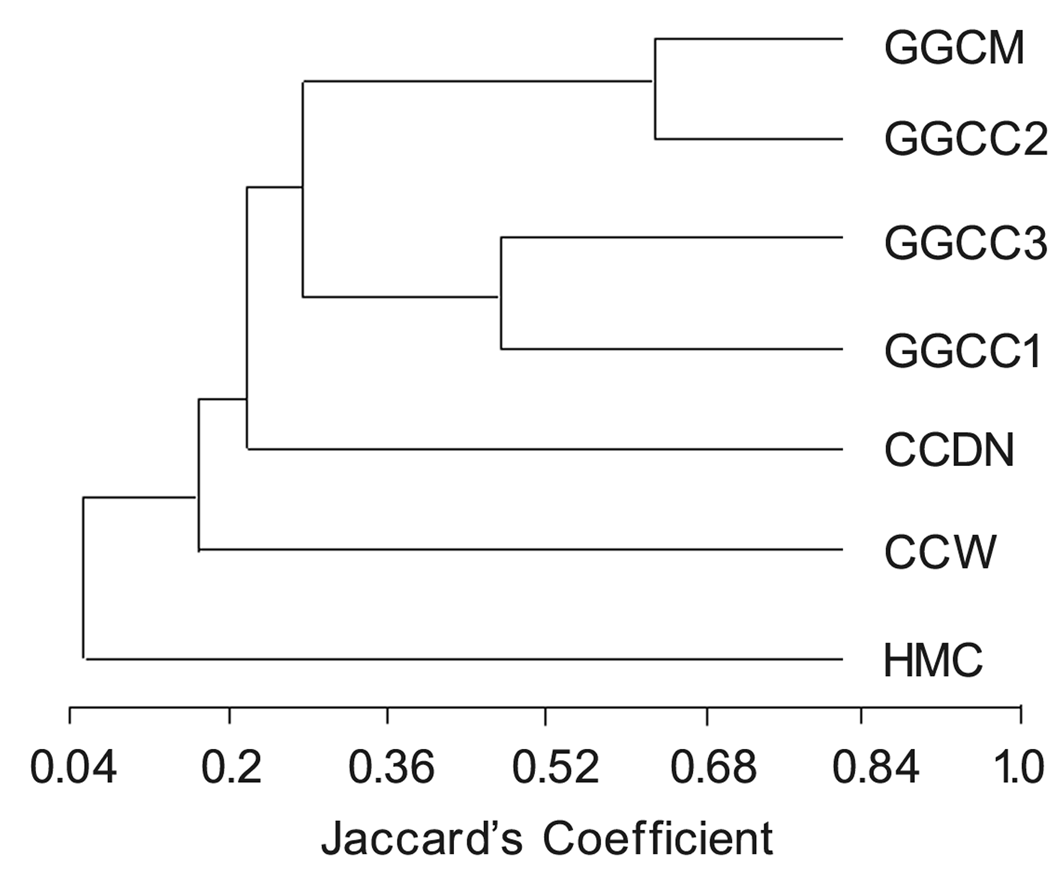

Supplement: Figure S3 — bacteria community similarity analysis. Comparison of bacteria community similarity based on DGGE fingerprint of 16S rRNA sequences. (TIF) [file pone.0030440.s003.tif]

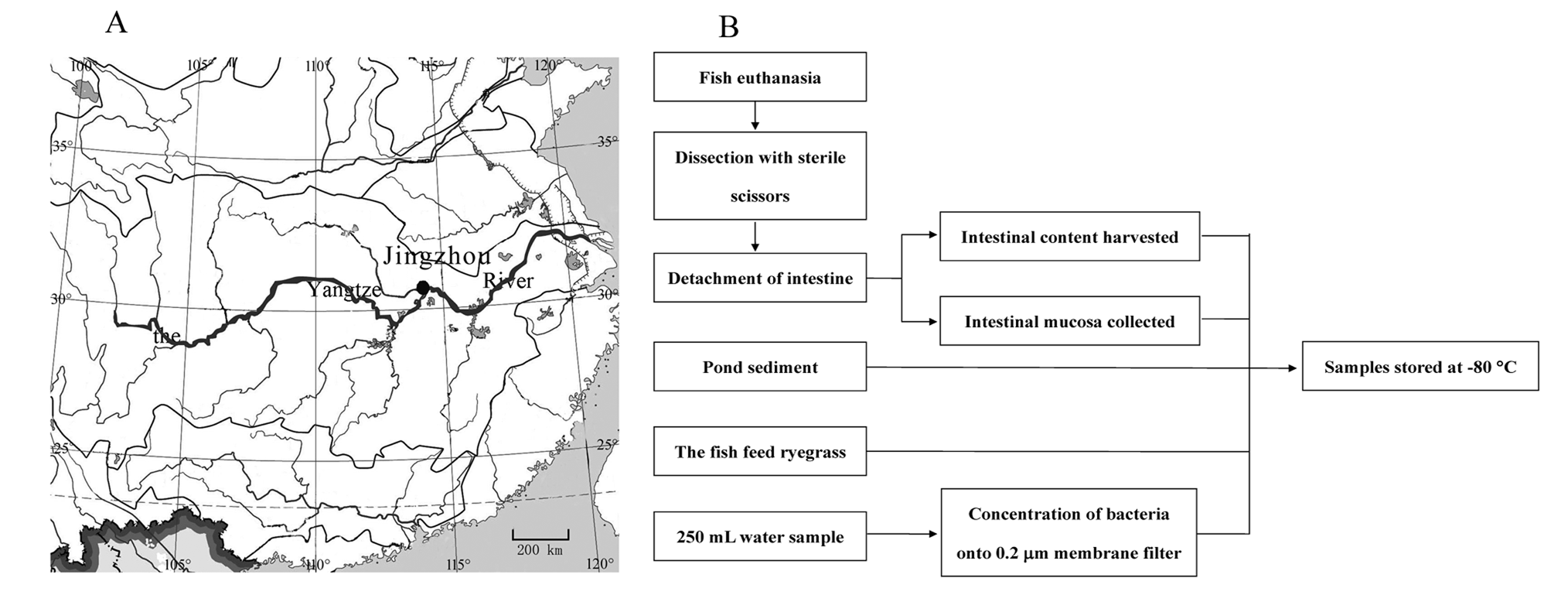

Supplement: Figure S4 — Sampling locality and procedures. Sampling locality and procedures in the present study. (A) Sampling locality, (B) Sampling procedures. (TIF) [file pone.0030440.s004.tif]
