# Supplementary material for: Composition, Diversity, and Origin of the Bacterial Community in Grass Carp Intestine
Source: PLoS One. 2012 Feb 20;7(2):e30440. doi: 10.1371/journal.pone.0030440 (PMC3282688; doi:10.1371/journal.pone.0030440)
Supplement: Table S1 — Classification of the 10 most abundant bacterial OTUs in the grass carp intestine contents and the associated environment, listed from most to least abundant. Relative abundance (%) of each OTU is included in parentheses. OTUs were identified using 97% cutoffs. GGCC1, GGCC2 and GGCC3 mean intestinal content of different individuals of grass carp. HMC, GGCM, CCDN, and CCW stand for grass carp feed ryegrass, intestinal mucosa of grass carp, pond sediment and pond water, respectively. (DOC) [file pone.0030440.s005.doc]

**Table S1.**

| CCDN | CCW | GGCC1 | GGCC2 | GGCC3 | GGCM | HMC |
| --- | --- | --- | --- | --- | --- | --- |
| *Prevotella* (5.51, OTU5746) | *Lactobacillus* (7.65, OTU2288) | *Veillonella* (12.57, OTU322) | Cyanobacteria (27.28,OTU13827) | *Veillonella* (8.28, OTU322) | Sphingobacteriales (24.70, OTU3771) | Cyanobacteria (66.19, OTU13827) |
| Fusobacteriales (4.74, OTU9262) | *Lactobacillus* (4.81, OTU2640) | Methylocystaceae (7.33, OTU14541) | *Rothia* (6.04, OTU8028) | Methylocystaceae (5.33, OTU14541) | *Clostridium* (12.96, OTU9919) | Cyanobacteria (5.13,  OTU14010) |
| *Veillonella* (4.53, OTU323) | *Flavobacterium* (3.87, OTU4493) | Cyanobacteria (5.70, OTU13827) | *Streptococcus* (5.37, OTU2136) | Cyanobacteria (5.11, OTU13827) | *Clostridium* (11.89, OTU9920) | *Veillonella* (4.09, OTU322) |
| *Veillonella* (4.13, OTU322) | *Candidatus*_*Planktophila* (3.22, OTU9801) | *Leuconostoc* (3.87, OTU2523) | *Veillonella* (4.40, OTU322) | *Leptotrichia* (4.71, OTU10110) | Sphingobacteriales (9.23, OTU4045) | *Actinomyces* (3.04, OTU4329) |
| Fusobacteriales (3.54, OTU8269) | *Lactobacillus* (2.43, OTU2431) | *Rothia* (3.83, OTU8028) | *Leuconostoc* (2.88, OTU2523) | *Anoxybacillus* (2.80, OTU2223) | *Leuconostoc* (7.48, OTU2523) | *Rothia* (2.3, OTU8028) |
| *Dechloromonas* (2.54, OTU4263) | *Limnohabitans* (1.89, OTU3669) | *Candidatus*_*Microthrix* (2.85, OTU10089) | *Pseudomonas* (2.69, OTU3954) | *Streptococcus* (2.74, OTU2136) | *Brevinema* (6.42, OTU6606) | *Actinomyces* (1.46, OTU4330) |
| Fusobacteriales (2.21, OTU9260) | *Polynucleobacter* (1.89, OTU4202) | *Uruburuella* (2.41, OTU4663) | Methylocystaceae (2.21, OTU14541) | *Anoxybacillus* (2.64, OTU2218) | *Brevinema* (2.60, OTU6911) | *Actinomyces* (1.07, OTU408) |
| Sinobacteraceae (1.81, OTU4224) | *Lactobacillus* (1.72, OTU2159) | *Nordella* (2.18, OTU14518) | *Stenotrophomonas* (2.12, OTU4165) | *Nordella* (2.11, OTU14518) | *Aeromonas* (1.82, OTU4626) | *Streptococcus* (1.04, OTU2042) |
| *Streptococcus* (1.59, OTU2042) | *Lactobacillus* (1.61, OTU2338) | *Citrobacter* (1.84, OTU3864) | Cyanobacteria (1.91, OTU14010) | *Leptotrichia* (1.95, OTU9695) | *Filibacter* (1.20, OTU2748) | Cyanobacteria (0.88, OTU13832) |
| Sinobacteraceae (0.95, OTU4645) | *Nordella* (1.55, OTU14518) | *Clostridium* (1.74, OTU9919) | *Prevotella* (1.63, OTU4289) | *Methyloversatilis* (1.80, OTU4243) | *Citrobacter* (1.09, OTU4554) | Cyanobacteria (0.79, OTU13885) |
