# Supplementary material for: Composition, Diversity, and Origin of the Bacterial Community in Grass Carp Intestine
Source: PLoS One. 2012 Feb 20;7(2):e30440. doi: 10.1371/journal.pone.0030440 (PMC3282688; doi:10.1371/journal.pone.0030440)
Supplement: Table S2 — Primer coverage. The primer sequences were compared with the RDP 16S rRNA gene sequence database to examine primer coverage using the Probe Match tool (http://rdp.cme.msu.edu/probematch/search.jsp) and exact matches were counted. 27F (used in this study), 5′-AGAGTTTGATCCTGGCTCAG-3′ 533R (used in this study), 5′- TTACCGCGGCTGCTGGCAC-3′ 534R, 5′-CAATTACCGCGGCTGCTGG-3′ 338R, 5′-TGCTGCCTCCCGTAGGAGT-3′ 338F, 5′-ACTCCTACGGGAGGCAGCAG-3′ 518R, 5′-ATTACCGCGGCTGCTGG-3′ 784F, 5′-AGGATTAGATACCCTGGTA-3′ 1061R, 5′-CRRCACGAGCTGACGAC-3′ (* R = A/G). (DOC) [file pone.0030440.s006.doc]

**Table S2.**

| Phylum/group | 27F | 338F | 784F | 338R | 518R | 533R | 534R | 1061R |
| --- | --- | --- | --- | --- | --- | --- | --- | --- |
| Firmicutes | 88940 | 405349 | 362034 | 405515 | 388618 | 388908 | 360377 | 303133 |
| Bacteroidetes | 35671 | 133086 | 125630 | 133144 | 140235 | 139776 | 135115 | 93072 |
| Actinobacteria | 17969 | 165867 | 154796 | 165921 | 163123 | 162853 | 117558 | 138050 |
| Proteobacteria | 105566 | 406815 | 382840 | 407038 | 413050 | 410371 | 393691 | 317437 |
| *Bacillus* | 4716 | 23492 | 20467 | 23498 | 23226 | 23158 | 22484 | 17332 |
| *Aeromonas* | 1166 | 4669 | 4114 | 4671 | 4454 | 4445 | 4325 | 3087 |
