# Supplementary material for: Composition, Diversity, and Origin of the Bacterial Community in Grass Carp Intestine
Source: PLoS One. 2012 Feb 20;7(2):e30440. doi: 10.1371/journal.pone.0030440 (PMC3282688; doi:10.1371/journal.pone.0030440)
Supplement: Text S1 — Protocol of PCR amplification, DGGE and T-RFLP analyses. (DOC) [file pone.0030440.s007.doc]

Supporting information

*PCR amplification and DGGE analysis*

The samples were initially subjected to DGGE analysis. 16S rRNA genes from different samples were amplified through nested PCR using the following general bacterial primer combinations: 27F (5′-AGAGTTTGATCCTGGCTCAG-3′)-1492R (5′-GGTTACCTTGTTACGACTT-3′) (primary) and 968F-1401R (secondary). For PCR-DGGE, a GC clamp was attached to the 5′-end of the 968F primer. The primary/secondary PCR reactions were performed on a TC-512 automated thermal cycler (Techne, UK) under the following cycling conditions: initial denaturation at 94 °C for 4 min, followed by 30 cycles of 30 s denaturation at 94 °C, 30 s annealing at 53 °C for primary and 57 °C for secondary PCR, and 90 s for primary and 40 s for secondary PCR elongation at 72 °C, and a final 6 min extension at 72 °C. These PCR amplifications were carried out in quadruplicate 25 mL reactions using the following reagents: ~5 ng bacterial genomic DNA or PCR products, 0.4 μM of each primer, 0.2 mM of each dNTP solution, 1 × PCR reaction buffer, 0.6 U of TaKaRa Ex Taq DNA polymerase (TaKaRa Corporation Ltd., Dalian, China), and double-distilled water to a final volume of 25 μL.

DGGE profiling was performed on a Dcode universal mutation detection system (Bio-Rad laboratories Inc., USA) according to the manufacturer's instructions. The PCR products were loaded onto 8% (w/v) polyacrylamide gels in 1× TAE buffer (40 mM Tris-HCl, 40 mM acetic acid, and 1 mM EDTA; pH 8.4). The polyacrylamide gels were prepared with a denaturing gradient ranging from 42% to 60% (100% denaturant contains 7 M urea and 40% formamide) to obtain the best discrimination between bacterial species. After electrophoresis, the gels were stained for 30 min with silver nitrate in TAE buffer, rinsed, and then photographed.

*PCR amplification and T-RFLP analysis*

For T-RFLP analysis, the PCR primers (27F and 1492R) were used; the amplification conditions are as described above. The primer 27F was fluorescently labeled on its 5′-end with carboxifluorescein (5′-/6-FAM), and Blend Taq-plus polymerase (Toyobo, Japan) was used instead of Ex Taq polymerase. The products from three independent amplifications were pooled for each sample. The pooled PCR products were digested with Mung bean nuclease (TaKaRa Corporation Ltd., China) to remove the single-stranded extensions. The digestion products were then purified using a DNA purification Kit (Axygen, China) according to manufacturer's recommendations.

Approximately 200 ng of the 16S rRNA gene amplification product from each sample was digested with restriction endonucleases *Hae*III (15 U), *Msp*I (10 U), or HhaI (10 U) (Fermentas, China) for 15 min at 37 °C. The digested fragments were purified using a DNA purification Kit (Axygen, China). The efficiency of restriction digestion was evaluated by agarose gel electrophoresis. Afterwards, the fluorescently labeled fragments were separated on a 3730 DNA Analyzer (Applied Biosystems, USA). The sizes of the fragments were determined by comparison with the internal GeneScan™ 500 LIZ® Si﻿ze Standard. Three independent replicate T-RFLPs were done for each 16S rRNA gene amplification.

The length of each terminal restriction fragment (TRF) sequence was visualized using GeneMapper® Software v4.1 (Applied Biosystems, USA). The following procedures were followed to obtain TRFs: the TRF sizes should lie in 50 bp and 500 bp, TRFs with fluorescent units (FU) not exceeding 200 were excluded, and two peaks were regarded as the same if the difference in the peak size was less than 1 nt. In addition, a 0.5% relative abundance threshold was applied, and the TRFs with areas less than the threshold were removed from the remaining analyses. Each unique TRF was considered an OTU. Only those TRFs presented twice in three independent replicate of each 16S rRNA gene amplification were considered valid TRFs.
